# Lentiviral vector–based xenograft tumors as candidate reference materials for detection of HER2-low breast cancer

**DOI:** 10.3389/fonc.2022.955943

**Published:** 2022-08-16

**Authors:** Yali Wei, Xu An, Qinmei Cao, Nanying Che, Yuanyuan Xue, Haiteng Deng, Qingtao Wang, Rui Zhou

**Affiliations:** ^1^ Department of Clinical Laboratory, Beijing Chaoyang Hospital, The Third Clinical Medical College of Capital Medical University, Beijing, China; ^2^ Department of Clinical Laboratory, Tongzhou Maternal and Child Health Hospital of Beijing, Beijing, China; ^3^ Department of Clinical Laboratory, China-Japan Friendship Hospital, Clinical Medical College of Capital Medical University, Beijing, China; ^4^ Department of Pathology, Beijing Chest Hospital, Capital Medical University, Beijing Tuberculosis and Thoracic Tumor Research Institute, Beijing, China; ^5^ Ministry of Education (MOE) Key Laboratory of Bioinformatics, Center for Synthetic and Systematic Biology, School of Life Sciences, Tsinghua University, Beijing, China

**Keywords:** HER2-low, lentiviral vector, digital PCR, reference materials, quality assurance

## Abstract

The human epidermal growth factor receptor 2 (HER2) is an important biomarker that plays a pivotal role in therapeutic decision-making for patients with breast cancer (BC). Patients with HER2-low BC can benefit from new HER2 targeted therapy. For ensuring the accurate and reproducible detection of HER2-low cancer, reliable reference materials are required for monitoring the sensitivity and specificity of detection assays. Herein, a lentiviral vector was used to transduce the HER2 gene into MDA-MB-231 cells that exhibited low HER2 density, and the cells were characterized by droplet digital PCR to accurately determine the copy number variation. Then, the formalin-fixed paraffin-embedded (FFPE) samples from xenografts were prepared and evaluated for suitability as candidate reference materials by immunohistochemistry (IHC) and fluorescence *in situ* hybridization (FISH). The FFPE reference materials were selected on the basis of IHC score of 2+ and negative FISH result to meet the requirement for HER2-low BC detection. Furthermore, the FFPE reference materials exhibited typical histological structures that resembled the clinical BC specimens. These novel FFPE reference materials displayed the high stability and homogeneity, and they were produced in high quantity. In summary, we generated high-quality reference materials for internal quality control and proficiency testing in HER2-low detection.

## Introduction

Breast cancer (BC) is one of the leading causes of cancer death in women (1). Human epidermal growth factor receptor 2 (HER2) is a well-known negative prognostic factor for BC and a target for the anti-HER2 therapy. Recently, antibody-drug conjugates (ADCs) have been applied to the ongoing clinical trials of HER2-low BC (2–7). On the basis of the latest guidelines of the American Society of Clinical Oncology (ASCO)/American College of Pathologists (CAP) for the HER2 detection method and scoring system (8), the HER2-low BC was defined as HER2 immunohistochemistry (IHC) 1+/2+ and fluorescence *in situ* hybridization (FISH) negative (9, 10). It has been shown that nearly 50% of patients with HER2-negative BC were actually classified as HER2-low, allowing them to benefit from the new anti-HER2 therapy (4). Therefore, it is important to accurately detect HER2 protein expression or gene amplification in patients with BC for maximizing therapeutic efficacy.

However, the accurate detection of HER2-low has been difficult for the following reasons. First, high genetic heterogeneity increases the difficulty of HER2 detection in BC (11, 12), causing ambiguous results over 90% of FISH detection (13). Moreover, there are controversial issues regarding to HER2 detection in BC diagnosis. According to the latest ASCO/CAP HER2 detection guidelines (8), IHC testing should be first performed. However, IHC testing was affected by many factors, such as tissue fixation (type and duration), antigen exposure, and antibody selection, which affect the reproducibility and repeatability. In addition, the positive immunostaining result of IHC testing was determined by semi-quantitative scores and is susceptible to considerable variability among these different observers. There are significant differences in the HER2 IHC scores, especially for cases with an IHC score of 2 + (14). The FISH testing was considered to be the gold standard for detecting HER2 gene amplification. It can quantitatively evaluate the copy number and ratio of the HER2 gene and the reference gene of chromosome 17 centromere (CEP17) of BC. However, this method is cumbersome and susceptible to the attenuation of fluorescent dye signals (15, 16). Recently, new HER2 detection methods have been proposed (17), such as a fully automated HER2 gene-protein assay (GPA), next-generation sequencing (NGS), or digital PCR (dPCR), and RNA FISH assay (RCasFISH) (18–21). The predictive performance of different detection methods varied (22, 23). On the basis of the ASCO/CAP 2018 guidelines, the standardization of laboratory procedures, data interpretation procedures, and internal and external quality control assessments was the key to generate reliable testing results.

The reference materials with distinct characteristics are used to design and verify testing methods in clinical laboratories and to evaluate laboratory performance by proficiency testing providers. Currently, there are two main types of HER2 reference materials: FFPE tumor tissues or tissue microarrays from clinical tumor patients (24, 25) and immortalized tumor cell lines (26, 27). For evaluating the performance of NGS in detecting HER2 copy number variation cell lines were used to extract genomic DNA (gDNA) at the National Institute of Standards and Technology (28), but it failed to meet the needs for monitoring the total testing process. For solving these issues, the FFPE materials, prepared by tumor cell lines and xenograft tumors, can produce typical histological structures similar to tumor tissues (29, 30). However, HER2-positive tumor cell lines have poor tumorigenic potential, which compromised its application (31). Therefore, it is important to establish a new type of HER2-low FFPE reference materials with distinct characteristics.

In the present study, the lentiviral vector system was used for constructing a HER2 overexpression cell line. Then, the droplet digital PCR (ddPCR) was used to screen the target monoclonal cell line to determine the ratio of HER2 and CEP17. Subsequently, the FFPE candidate reference materials were prepared by xenografting and were validated to meet the needs of clinical laboratory performance evaluation. The HER2-low FFPE reference materials are suitable for internal quality control and proficiency testing with existing HER2 detection methods.

## Materials and methods

### Construction of lentiviral vector–mediated cell line

#### Cell culture

Human embryonic kidney (HEK) 293T cells, MDA-MB-231 cells, and SK-BR-3 cells were purchased from the Cell Bank of the Chinese Academy of Sciences and cultured in Dulbecco’s modified Eagle’s medium (Wisent, Montreal, Canada), supplemented with 10% fetal bovine serum (Wisent, Montreal, Canada) and 1% penicillin and streptomycin (Wisent, Montreal, Canada). The cells were cultured at 37°C in an incubator containing 5% CO_2_.

#### Packaging and titration of recombinant lentivirus

**Figure 1:**
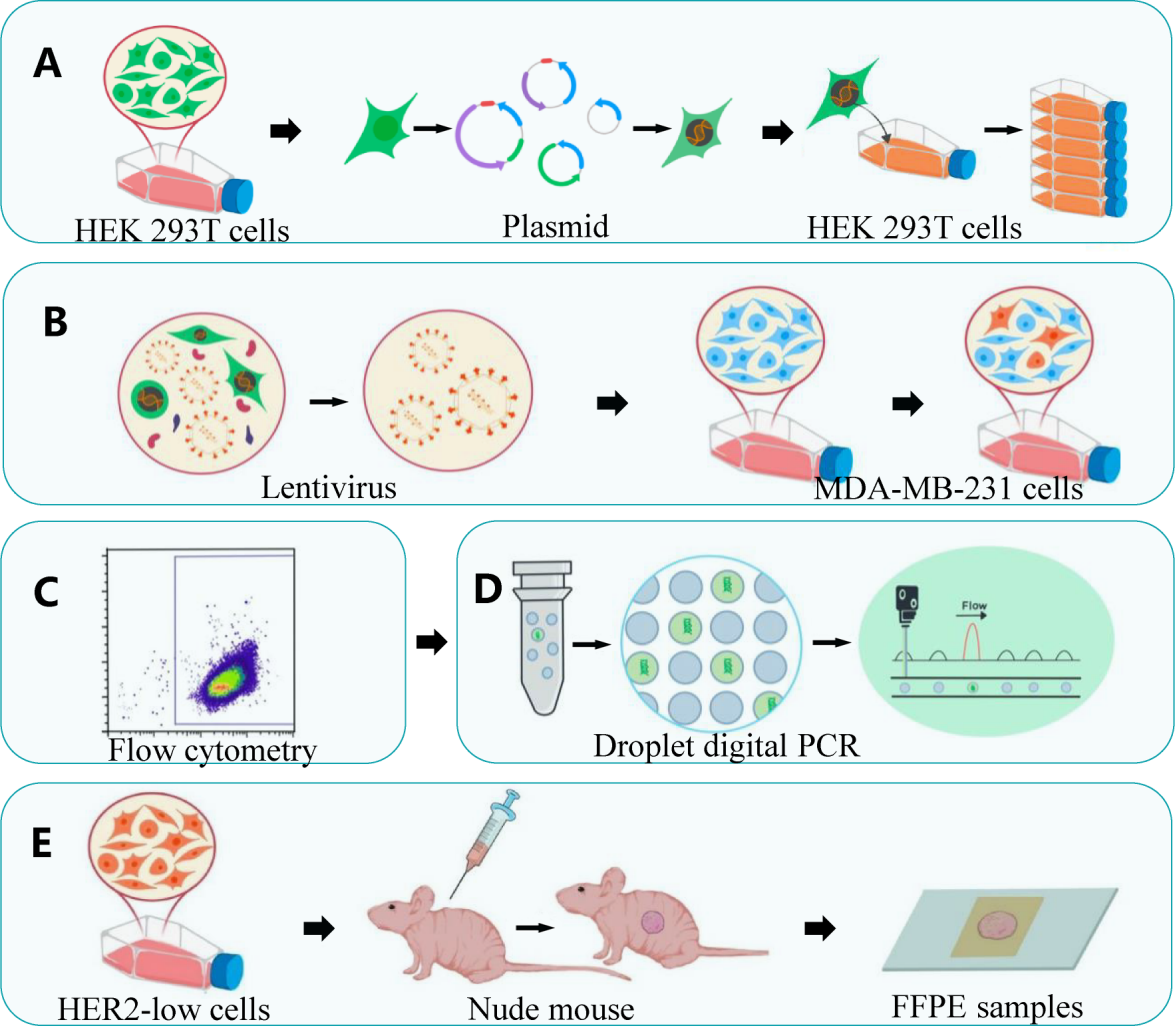
A schematic illustration of the generation of cell lines with HER2 expression *in vitro* and preparation of HER2-low FFPE reference material. **(A)** Recombinant lentivirus packaged and tittered in HEK 293T cells. **(B)** *In vitro* transduction in MDA-MB-231 cells **(C)** Detection of GFP expression by flow fluorescence scan **(D)** Verification the monoclonal cells using droplet digital PCR. **(E)** FFPE samples derived from xenograft tumors.

The pLVX-IRES-ZsGreen1 shuttle vector plasmid and helper vector plasmid (pLP2, pLP/VSVG, and pLP1) were purchased from American Type Culture Collection (ATCC) (Manassas, VA). The shuttle vector plasmid (pLVX-IRES-ZsGreen1-HER2), containing the target gene, was constructed by homologous terminal ligation method. The HEK 293T cells were mixed with pLVX-IRES-ZsGreen1-HER2 (20 µg), pLP2 (10 µg), pLP/VSVG (10 µg), pLP1 (15 µg), and transfection reagent PEI (48 µl), respectively (32). The medium was replaced with new complete medium after 6 h. Then, the lentivirus-rich cell supernatant was collected after 48 h of transfection and was concentrated with PEG6000 (TCI Shanghai, China). The lentivirus was collected and dispensed into sterile EP tubes for titer detection and stored in a refrigerator at −80°C for future use (**Figure 1A**).

#### Separation of lentivirus-infected cells by flow cytometry

Multiplicity of infection (MOI) values was set to 0, 5, 10, 20, and 50 in the preliminary experiment to determine the MOI value of lentivirus-infected MDA-MB-231 cells. When the growth density of MDA-MB-231 cells reached 30%, the cells were infected with concentrated virus solution and the co-transfection agent Polybrene (final concentration, 5–10 ng/µl; Sigma-Aldrich, USA) (**Figure 1B**). After 48 h of infection, the fluorescence of the cells was examined under a fluorescence microscope. Polyclonal cells were sorted by flow analyzer (FACSAria III, BD, USA) under aseptic conditions and sorted into 96-well plates with one cell per well according to fluorescence expression intensity (**Figure 1C**). After 2 weeks, the cells were examined under a microscope, and cells with uniform fluorescence were selected for amplification.

### Characterization of monoclonal cells by digital PCR, IHC, and FISH

#### Digital PCR

As an accurate nucleic acid quantification method approved by The Joint Committee for Traceability in Laboratory Medicine (JCTLM) (33), digital PCR required verification of the authenticity of nucleic acid quantification to determine the value of candidate reference materials. The national certified reference material (NCRM) NK603 was selected, which was a reference material certified by the National Institute of Metrology (https://www.ncrm.org.cn/). NCRM NK603 was a plasmid constructed with the sequence of a specific gene (NK603) and a reference gene (zSSIIb). The NCRM NK603 plasmid was quantified by the digital PCR procedure according to manufacturer’s instructions. To ensure the traceability of this plasmid measurement, the measuring instrument and procedure were in accordance with the measurement metrological characteristics. The primers and probes were designed according to the instructions (**Table 1**). The copy number of NCRM NK603 standard and the ratio of the NK603 gene to the reference gene were determined after dilution according to the instructions. The minimum information dMIQE2020 guidelines for publishing digital PCR experiments were followed during trial development and sample measurement (33).

**Table 1:** PCR assays primers used for certification values.

	Primer name	Sequence (5′ to 3′)	PCR amplicon
HER2 amplification	HER2-F	CTCATCGCTCACAACCAAGT	112 bp
	HER2-R	GGTCTCCATTGTCTAGCACG	
	HER2-P	FAM-ACCCAGCTCTTTGAGGACAACTATGC-BHQ1	
	CEP17-F	GCTGATGATCATAAAGCCACAGGTA	81bp
	CEP17-R	TGGTGCTCAGGCAGTGC	
	CEP17-P	VIC-TGCTGCAATAGGCGG-BHQ1	
NCRM GBW10086	NK603-F	ATGAATGACCTCGAGTAAGCTTGTTAA	108bp
	NK603-R	AAGAGATAACAGGATCCACTCAAACACT	
	NK603-P	FAM-TGGTACCACGCGACAGACTTCCACTC-BHQ1	
	zSSIIb-F	CTCCCAATCCTTTGACATCTGC	151bp
	zSSIIb-R	TCGATTTCTCTCTTGGTGACAGG	
	zSSIIb-P	VIC-AGCAAAGTCAGAGCGCTGCAATGCA-BHQ1	

F, forward primer; R, reverse primer; P, TaqMan fluorescent probe; HER2, human epidermal growth factor receptor 2; CEP17, chromosome 17 centromere; NK603, specific gene of NCRM; zSSIIb, reference gene of NCRM.

The monoclonal cells were cultured and digested by trypsin/EDTA (Wisent, Montreal, Canada). gDNA was extracted using the QIAamp DNA Mini Kit (Qiagen, Hilden, Germany) to ensure a minimum of 20–30 ng of DNA as nucleic acid input. Subsequently, the validated ddPCR was used to screen monoclonal cell lines (**Figure 1D**). Experimental procedures were based on the manufacturer’s instructions, and all of consumables were purchased from Bio-Rad. The reaction was performed with a 20-µl reaction volume, including the extracted DNA (2 µl), the probe’s 2× ddPCR supermix (10 µl), 20 µM HER2 forward primer (0.9 µl), 20 µM HER2 reverse primer (0.9 µl), HER2 FAM probe (0.25 μl), 20 μM CEP17 forward primer (0.9 μl), 20 μM CEP17 reverse primer (0.9 μl), CEP17 VIC probe (0.25 μl), and deionized distilled water (3.9 μl). The primers and probes were designed on the basis of the published protocols (34, 35), as shown in **Table 1**. The DNA reaction solution containing the samples and 70 µl of oil-producing droplets was placed on a DG8 plate, and then, the nanoscale oil-in-water droplets were generated on the plate by a droplet generator (QX200, Bio-Rad, USA). The droplets were transferred to a 96-well PCR plate, and then, the PCR plate was sealed with aluminum foil heat seal film (PX1, Bio-Rad, USA) and placed in a thermal circulator (C1000, Bio-Rad, USA). Thermal cycling conditions were the followings: 95°C for 10 min, 94°C for 30 s, 60°C for 60 s, 98°C for 10 min, 4°C hold, 40 cycles. Then, the PCR plate was transferred to a droplet reader (QX200, Bio-Rad, USA), and the data were collected and analyzed by a software package provided by dPCR (QuantaSoft 1.7.4, Bio-Rad, USA).

In addition, gDNA was extracted from peripheral blood mononuclear cell (PBMC) by a QIAamp DNA Mini Kit (Qiagen, Hilden, Germany) and was used as external quality control. Similarly, gDNA was extracted from MDA-MB-231 cells as HER2 negative control and SK-BR-3 cells as HER2-positive control. The measurements were repeated six times for each sample and the average value was calculated.

#### Cell-slide IHC detection

The assigned monoclonal cells were immobilized on polylysine slides fixed with 4% paraformaldehyde (Solarbio, Beijing, China) for 20 min. Subsequently, the cell slide was blocked with 10% goat serum, and it was incubated with prediluted with rabbit anti-HER2 monoclonal antibody 4B5 monoclonal antibody (prediluted; Ventana, Tucson, AZ, USA) at 37°C for 1 h, and then, it was manually stained with Rabbit IHC Kit (Beijing 4A Biotechnology Co., Ltd., Beijing, China). The cell slide was counterstained with hematoxylin, incubated with blue reagent, and observed with a microscope for color development. SK-BR-3 and MDA-MB-231 cell slides were used as positive and negative controls, respectively.

#### Cell-slide FISH detection

Similarly, cell slides were prepared by baking at 65°C for 30 min. Then, pepsin solution was added, and HER2 amplification was assessed using a dual fluorescence kit PathVysion (Abbott Vysis Probes, Chicago, IL). All procedures were carried out in accordance with the manufacturer’s instructions. The probe was denatured (73°C, 3 min) and hybridized (37°C, 17 h) on slides. All sample slides were washed in the dark after hybridization with washing buffer supplied by the manufacturer. 4,6-diamino-2-phenyl indole (DAPI) reverse staining was performed on the slide target area, and red and green fluorescence in 20 cells were visually detected by fluorescence microscope. According to the instructions, the results of sample slides were qualitatively evaluated. Sk-BR-3 cell slides and MDA-MB-231 cell slides were used as positive control and negative control, respectively.

### The establishment of xenotransplantation model

Female BALB/c nude mice, aged 6 weeks, were purchased from Vital River (Beijing, People’s Republic of China). The nude mice weighed 18 to 20 g and were kept in the specific pathogen–free animal room. Animal experiments were approved by the Animal Feeding and Use Committee of Tsinghua University, and they were carried out in accordance with the relevant regulations on animals. The diluted and validated 1 × 10^7^ HER2-low cells were subcutaneously injected into the axilla of mice (**Figure 1E**). Xenograft tumors were measured weekly with vernier calipers. When the transplanted tumor size reached about 1,000 mm^3^, the mice were sacrificed by cervical dislocation. Then, the transplanted tumor was fixed in 10% neutral buffer overnight with formalin. Subsequently, the German Leica paraffin embedding machine was used for automatic tissue embedding. Each sample was cut into 5-μm-thick slices (**Figure 1E**). HE staining was used to observe the histological and cellular morphology of each FFPE tumor tissue. The tumor area and histological structure were evaluated by a pathologist.

### Validation of FFPE reference materials

#### FFPE for IHC detection

Compared with cell slides, FFPE samples were fully dewaxed with xylene, hydrated with gradient alcohol, repaired with antigen recovery solution (ZSGB-BIO, Beijing, China; pH 8.0) in high-pressure heat, and digested with pepsin at room temperature for 10 min. The remaining steps were the same as above. The IHC staining scores were calculated on the basis of the 2018 ASCO/CAP HER2 update guideline (8).

#### FFPE for FISH detection

The dual fluorescence kit PathVysion (Abbott Vysis Probes, Abbott Laboratories, Chicago, IL) was also used to evaluate the HER2 amplification in FFPE. All procedures were carried out in accordance with the manufacturer’s instructions.

#### Assessment of compliance with clinical specimens

Four surgical specimens of invasive BC with typical HER2 expression, confirmed by IHC, were collected and approved by the Ethics Committee of Chest Hospital Affiliated to Capital Medical University. IHC and FISH tests were performed separately for each sample. The procedure was same as abovementioned methods. The HER2 expression of these four samples was evaluated together with the novel reference materials.

#### Assessment of uniformity and stability

Both FISH and IHC were employed in qualitative analysis, whereas ddPCR was also used to identify HER2-low FFPE reference materials in this study. The paraffin-embedded wax block was divided into 50 individual parts (10 μm thick). Ten sections were randomly selected, and gDNA was extracted by the QIAamp DNA FFPE tissue kit (Qiagen, Hilden, Germany) for ddPCR detection. The analysis of 10 groups of samples was performed over the entire sample sequence. The samples were stored at 37°C, ambient temperature, and 4°C for 1 week, 2 weeks, 1 month, and 2 months, respectively, and all experiments were repeated three times. Then, gDNA was extracted to verify the actual stability of the FFPE reference materials.

## Results

### Overexpression of HER2 using lentivirus vector system

**Figure 2:**
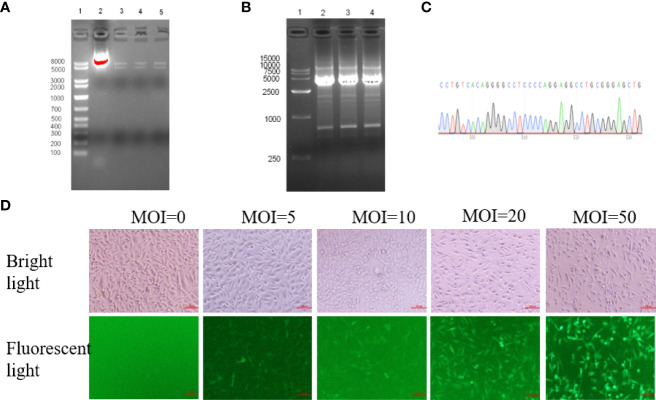
HER2 overexpression was generated in MDA-MB-231 cells using a lentivirus vector system. **(A)** After double restriction endonuclease Sph1 and Nhe1 digestion, electrophoresis showed two specific target bands of about 5kbp and 7kbp, line 1: Marker; line 2: Recombinant plasmid without enzyme digestion; line 3-5: Double digestion with three replicates. **(B)** The positive colonies of extracted plasmid were selected for PCR identification, and agarose gel electrophoresis showed a specific target band of about 3.7kbp. **(C)** The sequences were validated by sanger sequencing(Partial display. **(D)** MDA-MB-231 cells were transduced by lentivirus vector at five different multiplicity of infection (MOI 0, 5, 10, 20 and 50 virus particles per cell). GFP expression in cells was analyzed by flow cytometry 48 hours after lentivirus vector infection (×100).

First, the HER2-cDNA plasmid was connected to pLVX-IRES-ZsGreen1 plasmid by the homologous terminal recombination method. The recombinant plasmid was cut with restriction endonuclease Sph1 and Nhe1 and separated by Agarose electrophoresis, generating two specific target bands of 5 and 7 kbp as expected (**Figure 2A**). After PCR reaction, agarose gel electrophoresis of the PCR product revealed specific target bands of about 3.7 kbp (**Figure 2B**). The PCR products were sent to Ruibio Biotechnology Co., Ltd., Beijing for Sanger sequencing, and the sequencing result showed that it was completely consistent with the BLAST sequence of HER2 in GenBank (**Figure 2C**). These results confirmed that the eukaryotic expression vector pLVX-IRES-ZsGreen1-HER2 plasmid was successfully constructed.

Then, HEK 293T cells were transfected to produce lentiviruses as described in the method section. Lentiviral vectors were transduced into MDA-MB-231 cells at five different infection multiples (MOI of 0, 5, 10, 20, and 50 virus particles per cell), respectively. Green Fluorescent Protein (GFP) expression was observed 48 h after infection (**Figure 2D**; 100×). Cells with different fluorescence intensity were sorted by flow cytometry for single-cell clones with GFP fluorescence (**Supplementary Figure S1**). Therefore, lentiviruses were successfully transfected into MDA-MB-231 host cells and expressed stably in the host cells. Monoclonal cell lines were cultured for further validation.

### Identification and validation of the HER2-low cells

NCRM NK603 plasmid was selected as the trueness verification sample to investigate the accuracy of the digital PCR method. The given standard value of NCRM NK603 plasmid is 2.40 × 10^8^ copy/μl, and the expanded uncertainty (k = 2) is 0.14 × 10^8^ copy/μl. According to the instructions, NCRM NK603 was diluted to an appropriate concentration in this study, and the quantitative average value was 2.37 × 10^8^ copy/μl by digital PCR, which was within the allowable uncertainty range. The given ratio of the NK603-specific gene fragment to the internal reference gene (zSSIIb) is 0.97, and the expanded uncertainty (k = 2) is 0.09. We determined the ratio of NK603/zSSIIb by digital PCR to be 1.02, which was within the allowable uncertainty. The results showed that the experimental method satisfied the quantitative requirements of metrology traceability in this study.

**Figure 3:**
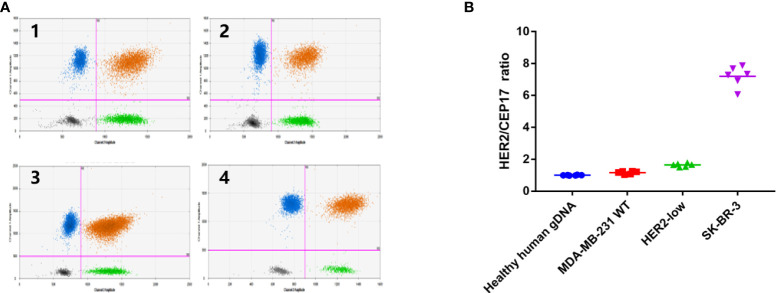
Monoclonal cultured cells assigned by ddPCR. **(A)** Data was shown in the form of 2D amplitude of Bio-Rad droplet digital PCR. The number of microdroplet generated by all reactions was more than 10000, in accordance with Poisson distribution statistical principle, and the fluorescence of FAM (HER2) and VIC (CEP17) was clearly distinguished. і:Healthy human gDNA; ii: MDA-MB-231 WT cells; iii: HER2-low cells; iv: SK-BR-3 cells, **(B)** Data shown in the form of ratio (HER2/CEP17). The HER2/CEP17) ratios of healthy human genomic DNA, MDA-MB-231 cell line, HER2-low cells and SK-BR-3 cell line were 1.001 (0.982-1.026), 1.1638 (1.033-1.254), 1.693(1.632-1.775) 7.194 (6.066-7.872).

Then, experimental conditions were optimized to examine the HER2-low monoclonal cells. The number of droplets generated by all reactions exceeded 10,000, which conformed to the statistical principle of Poisson distribution. The FAM fluorescence and VIC fluorescence were clearly distinguished (**Figure 3A**). The average HER2/CEP17 ratios of healthy human gDNA, MDA-MB-231 cell line, HER2-low cell line, and SK-BR-3 cell line were 1.001 (0.982–1.026), 1.1638 (1.033–1.254), 1.693 (1.632–1.775), and 7.194 (6.066–7.872), respectively. The coefficient of variation of HER2/CEP17 ratio of HER2-low monoclonal cells was CV < 10% (**Figure 3B**). These results confirmed that HER2-low monoclonal cells were suitable for the xenotransplantation experiment in nude mice.

**Figure 4:**
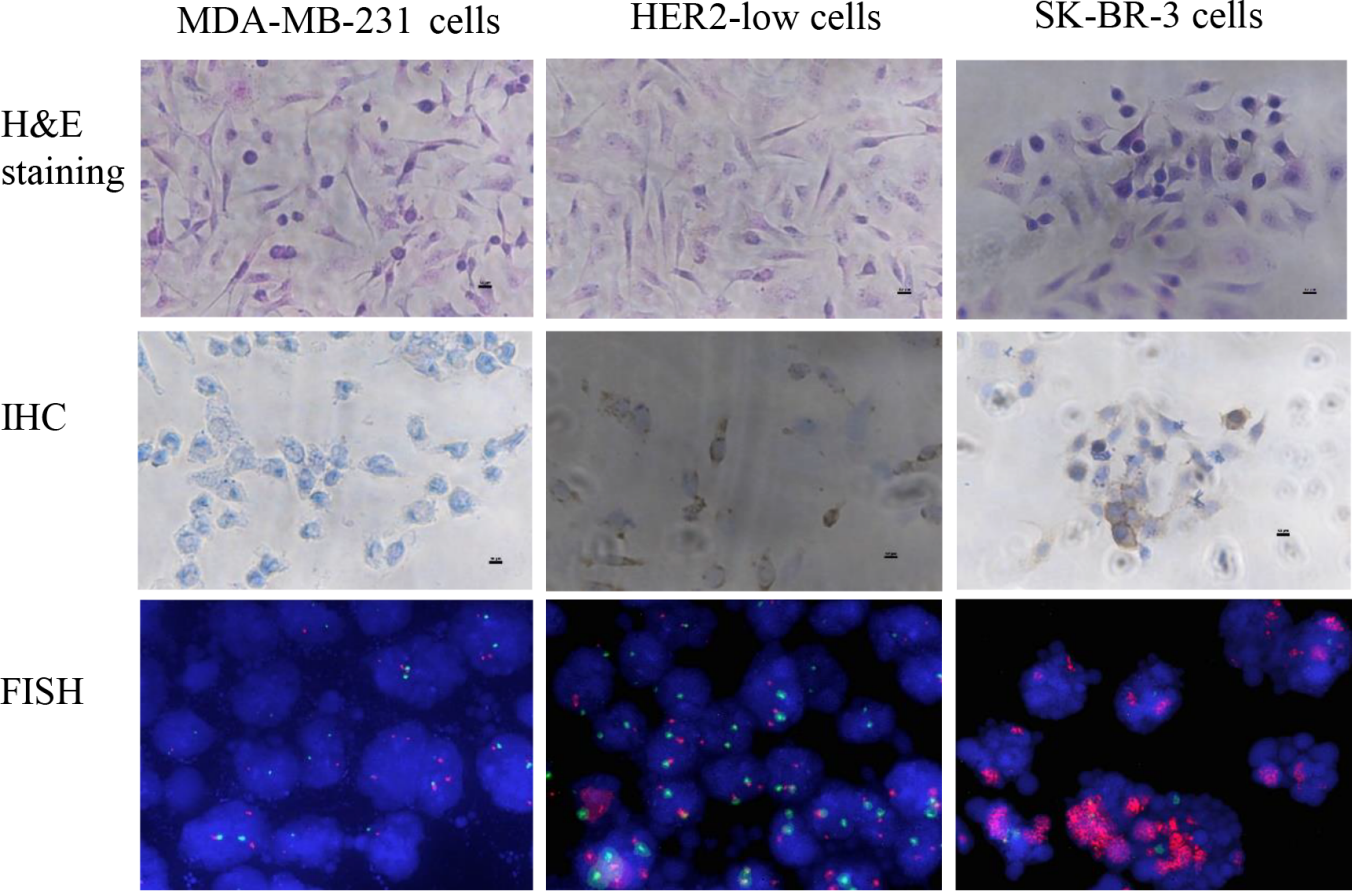
The cell slide evaluated that HER2-low cells can be detected by conventional detection methods. HE staining showed that the cell morphology was intact. IHC showed that MDA-MB-231 WT cell line had no staining and was negative. SK-BR-3 was brown-yellow, +++. HER2-low was moderately stained, denoted as ++. FISH showed that MDA-MB-231 WT cells had no amplification of HER2 (HER/CEP17 ratio was 1.057); the HER2 signal in SK-BR-3 was clustered and significantly amplified; HER2-low cells had no amplification of HER2 (HER/CEP17 ratio was 1.375).

Cell slides were prepared to verify that HER2-low was detected by conventional assays. The HE staining showed that the cell morphology was intact (**Figure 4**). In addition, the IHC results showed that the MDA-MB-231 cells were HER2 negative, whereas SK-BR-3 cells were brownish yellow marked as +++. HER2-low cells were moderately stained as marked as ++ (**Figure 4**). The FISH results showed no amplified HER2 gene with a ratio of HER/CEP17 as 1.057 in MDA-MB-231 cells and amplified HER2 gene in SK-BR-3 cells. There were no amplified HER2 genes in HER2-low cells with the ratio of HER/CEP17 1.375 (**Figure 4**). These results demonstrated that the HER2-low cells fully satisfied the clinical requirements, in which IHC was 1+/2+ and FISH was negative.

### Characteristics of the FFPE reference materials

**Figure 5:**
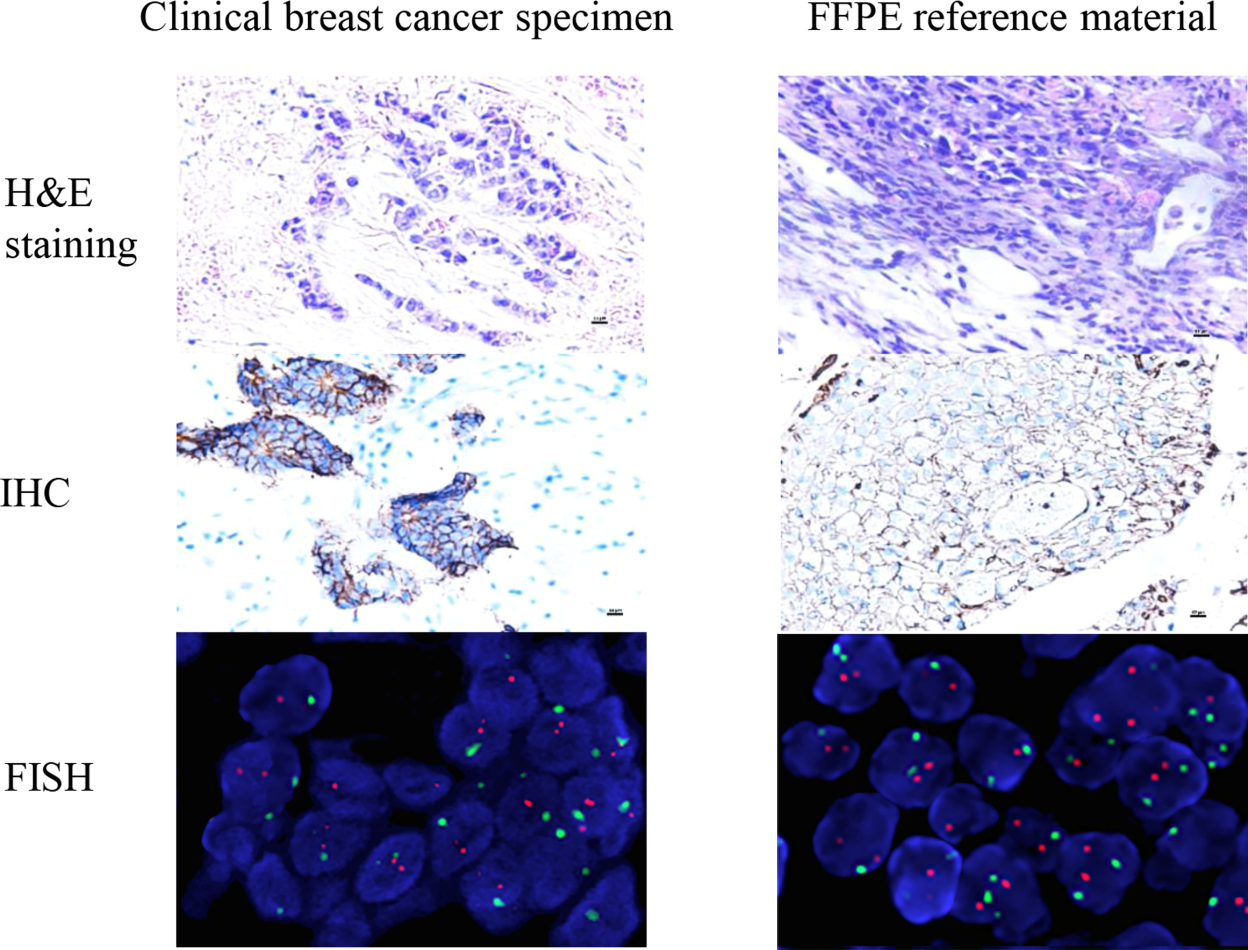
The characteristics of the reference materials were evaluated for conformity with the real clinical specimens. The histopathological structure of the xenotransplantation was highly consistent with the real clinical specimens. The real clinical specimen was showed IHC 2+ and FISH negative. The results show that the candidate reference material fully meets the clinical needs of HER2-low (IHC 1+/2+ and FISH negative).

When 1 × 10^7^ HER2-low cells were injected into the axilla of BALb/c nude mice, the transplanted tumor grew to 100 mm^3^ after 4 weeks. Then, the tumor tissue was surgically removed and placed in 4% neutral formaldehyde. The tumor tissue was fixed by formaldehyde and embedded in paraffin and excised into 5-μm wax rolls. The wax rolls were mounted on glass slides that were prepared for FFPE reference materials. The characteristics of the reference materials were evaluated for conformity with the real clinical BC specimens, in which we found that the typical histological structures of FFPE reference materials derived from xenografted tumors were similar to those derived from BC tumors in humans. The percentage of tumor cells on each FFPE reference material was more than 80% in the HE staining, as shown in **Figure 5**.

### Assay validation and commutability assessment of the FFPE reference materials

To verify whether reference materials resembled the clinical specimens, we used IHC and FISH to analyze the FFPE reference materials. The IHC results showed that reference materials were moderately stained as marked as 2+. Using FISH fluorescent staining, the FFPE reference materials were examined under low magnification to determine the detection quality and the heterogeneity of HER2 gene amplification. Dichroic signals were counted in 20 tumor cells randomly and showed that HER2/CEP17 ratio was smaller than 2.0 (1.147). The results of IHC 2+ and FISH negative were in agreement with those from tumor cells, satisfying the HER2-low requirements (**Figure 5**). The IHC scores of the clinical specimens were determined at 0, 1+, 2+, and 3+ (**Supplementary Figure S2**). The histopathological structure of the FFPE reference materials was highly resembled those of the clinical specimens (**Figure 5**).

### Homogeneity and stability assessment of the FFPE reference materials

**Figure 6:**
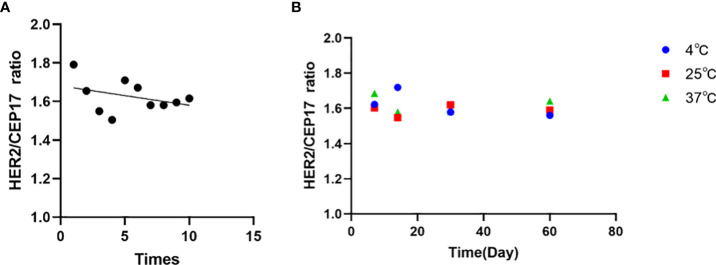
Sample validation and homogeneity and stability assessment of the FFPE reference materials by ddPCR. **(A)** Sample validation by ddPCR of the FFPE reference materials. Data shown in the form of ratio (HER2/CEP17). **(B)** Homogeneity and stability assessment of the FFPE reference materials showing that no significant trends in the ratio are detectable within the detection limits of the assays. The mean values of all samples are consistent with their individual target values.

To examine the homogeneity and stability of the FFPE reference materials, 10-section FFPE reference materials were randomly selected for ddPCR detection. On the basis of one-way ANOVA with P > 0.05, our results indicated that there was no significant difference among samples, and the samples were highly uniform (**Figure 6A**). No significant differences were observed for the reference materials stored at 37°C, ambient temperature, and 4°C for 1 week, 2 weeks, 1 month, and 2 months. Moreover, the average values of FFPE samples were consistent with those in target cells with deviation <10.0% (**Figure 6B**). Therefore, we concluded that the FFPE reference materials were completely homogeneous and they were stable for at least 2 months.

## Discussions

BC is the most commonly diagnosed malignant tumor and is the leading cause of cancer deaths among women worldwide (1) and HER2-positive BC tumors are characterized with strong aggressiveness and poor prognosis. It is generally agreed that the patients with 3+ of HER2 detected by IHC are considered as HER2 overexpression. Similarly, the patients with 2+ of HER2 detected by IHC and positive gene amplification by FISH are also considered as HER2 positive (8). HER2-positive breasts cancer accounts for approximately 15%–20% of BC. It has been demonstrated that the HER2-low patients with IHC0/1+/2+ and FISH negative exhibited different biological and clinical prognostic characteristics from those HER2-positive patients. Novel HER2-directed ADCs have showed efficacy in treating HER2-low patients with IHC scores of 1+ or 2+ and with FISH negative (9).

Reference materials are important for ensuring comparability of results. Clinical specimens are not the high-quality reference materials for high heterogeneity. On the other hand, reference materials made by paraffin embedding of human tumor cells or xenograft tissue were rapidly developed over the last decade (36, 37).

The widely used BC cells cannot satisfy the HER2 IHC1+/2+ and FISH-negative requirements for HER2-low diagnosis (26), and they exhibit poor tumorigenicity and are difficult to form xenograft tumors (31). In the present study, MDA-MB-231 BC cells with strong tumorigenicity were selected to establish a stable cell line, in which HER2 gene was overexpressed using lentiviral vector, which was demonstrated as a gene delivery vector with unique versatility and robustness (38) for long-term expression of transgenes *in vitro* and *in vivo* (32).

The stable cell lines in which HER2 were overexpressed were characterized by the digital PCR technology, which reliably and accurately quantified nucleic acid target sequences without calibration, and determined the absolute copy number and nucleic acid content. Because of its high precision and high reproducibility, digital PCR technology can be applied for clinical diagnosis and quantified for reference materials (39, 40). In this study, the Chinese national standard material was used to verify the accuracy of digital PCR procedure in this study. Subsequently, the nucleic acid copy number of HER2 overexpressed cell line was quantified by verified digital PCR procedure. The quantitative results of digital PCR were consistent with those of conventional FISH.

The HER2-overexpressing cells were employed to establish xenograft tumors in nude mice. We demonstrated that the FFPE reference materials from xenograft tumors exhibited the complete histopathological structure, high homogeneity, and stability, highly resembling the actual clinical specimens of BC. In addition, the candidate FFPE reference materials showed consistent IHC and FISH results, matching to those classified HER2-low clinical specimens.

In summary, we developed reliable reference materials based on lentiviral vector–mediated gene-overexpression and xenograft to ensure the accuracy and reproducibility of HER2-low detection. We demonstrated that the FFPE reference materials are in high-quality assurance to meet the standard analytical performance and are suitable for internal quality control and proficiency testing of HER2-low detection.

## Data availability statement

The original contributions presented in the study are included in the article/**Supplementary Material**. Further inquiries can be directed to the corresponding author/s.

## Ethics statement

This study was reviewed and approved by The Animal Feeding and Use Committee of Tsinghua University. Written informed consent was obtained from the individual(s) for the publication of any potentially identifiable images or data included in this article.

## Author contributions

YW, XA, QC performed this experiment. YX assisted in data analysis. QW and HD designed the research. YW and RZ wrote the manuscript. NC provided after-testing clinical samples. All authors contributed to the article and approved the submitted version.

## Funding

This study was supported by the Beijing Municipal Administration of Hospitals Clinical Medicine Development of Special Funding Support (ZYLX201811); and the Excellence Project of key clinical specialty in Beijing; and the 1351 Talent Training Plan [grant numbers CYMY-2017-01].

## Conflict of interest

The authors declare that the research was conducted in the absence of any commercial or financial relationships that could be construed as a potential conflict of interest.

## Publisher’s note

All claims expressed in this article are solely those of the authors and do not necessarily represent those of their affiliated organizations, or those of the publisher, the editors and the reviewers. Any product that may be evaluated in this article, or claim that may be made by its manufacturer, is not guaranteed or endorsed by the publisher.
