# Supplementary material for: Lentiviral vector–based xenograft tumors as candidate reference materials for detection of HER2-low breast cancer
Source: Front Oncol. 2022 Aug 16;12:955943. doi: 10.3389/fonc.2022.955943 (PMC9425432; doi:10.3389/fonc.2022.955943)

**Supplement Figure1** Cells with different fluorescence intensities were sorted by flow cytometry for single-cell clones with GFP fluorescence.

**
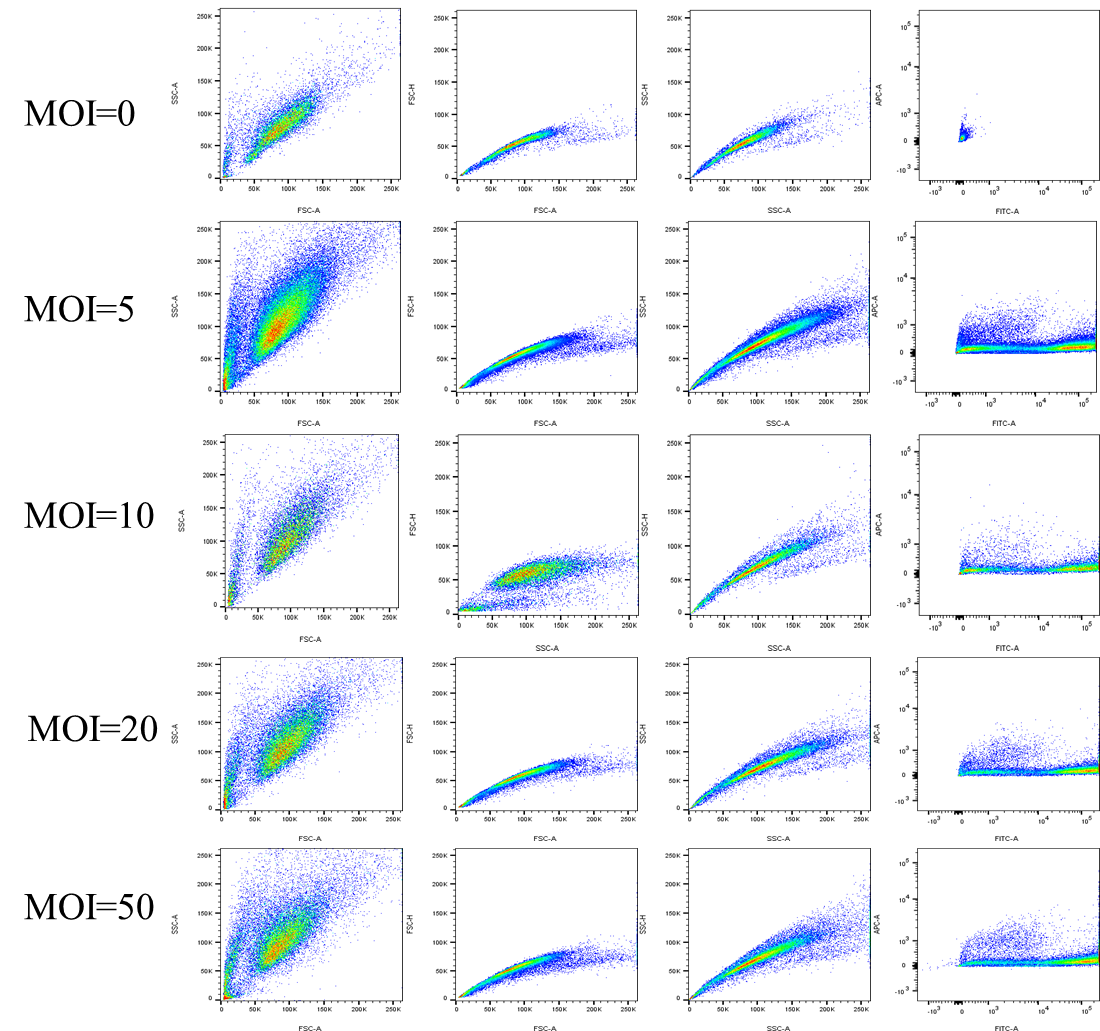
**

**Supplement Figure2** The 4 surgical specimens of invasive breast cancer with typical HER2 status tested by IHC were collected. The IHC scores of the real specimens were 0, 1+, 2+, and 3+; FISH detection was performed on each sample separately and the results were negative, negative, negative, positive.


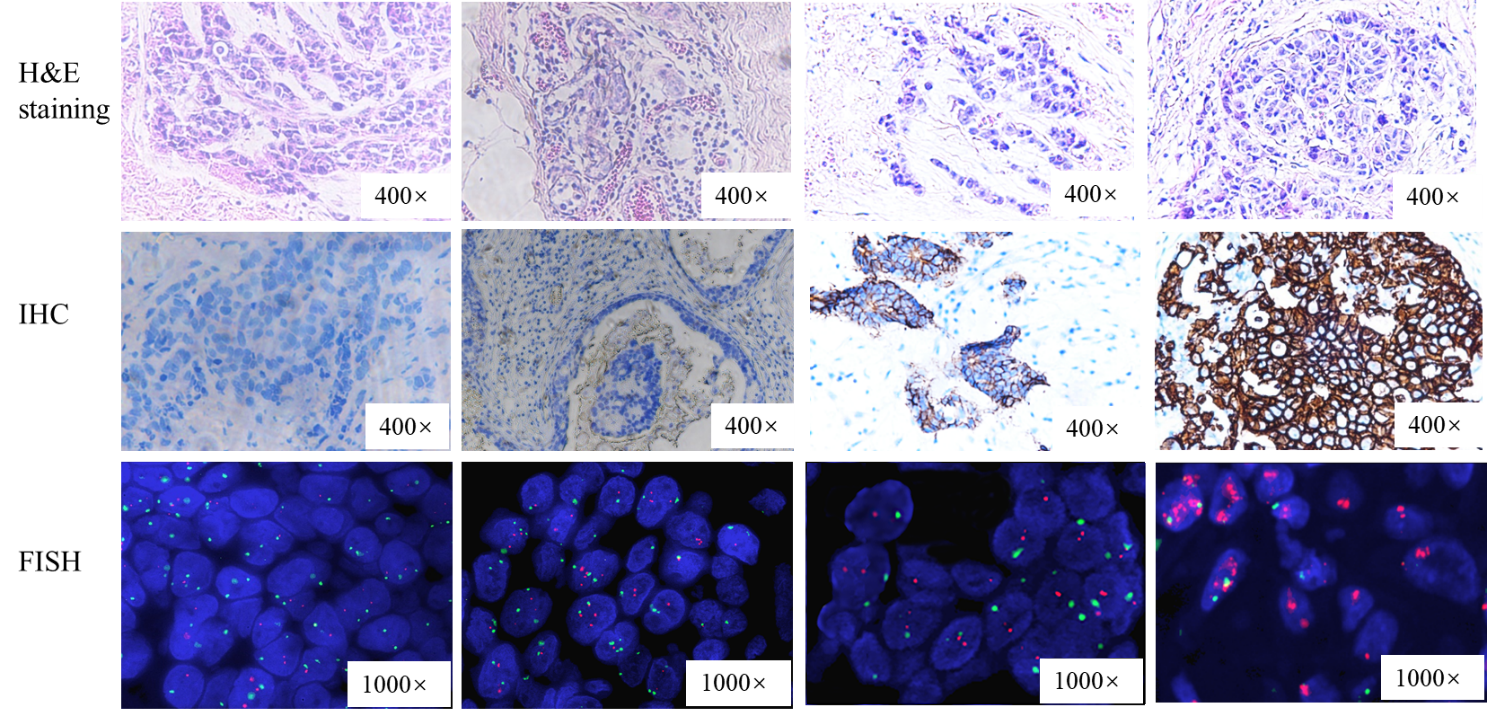

Supplement: Supplementary file 1 [file DataSheet_1.docx]
